# Cutaneous involvement in diffuse large B cell lymphoma at presentation: report of two rare cases and literature review

**DOI:** 10.1186/s43046-021-00085-1

**Published:** 2021-09-13

**Authors:** Sindhu Kilaru, Soumya Surath Panda, Sourav Mishra, Debahuti Mohapatra, Manas Baisakh, Spoorthy Kolluri, Suma Devaraj, Lalatendu Moharana, Ghanashyam Biswas

**Affiliations:** 1 Department of Medical Oncology, IMS and SUM Hospital, Siksha ‘O’ Anusandhan University, Bhubaneshwar, Odisha India; 2 Department of Medical Oncology, Apollo Hospital, Bhubaneswar, Odisha India; 3 Department of Pathology, IMS & SUM Hospital, Siksha ‘O’ Anusandhan University, Bhubaneswar, Odisha India; 4 Department of Pathology, Apollo Hospital, Bhubaneswar, Odisha India; 5 Department of Medical Oncology, Sparsh Hospital, Bhubaneswar, Odisha India

**Keywords:** Lymphoma, Diffuse large B cell lymphoma, Cutaneous lymphoma, Ulcer, ki-67

## Abstract

**Background:**

Diffuse large B cell lymphoma (DLBCL) can occur at nodal and/or extra-nodal sites. After the gastrointestinal tract, cutaneous involvement predominates in extra-nodal DLBCL. Skin involvement at presentation can be in the form of plaques, papules, nodules or ulcers. Differentiating primary cutaneous DLBCL from systemic DLBCL with cutaneous involvement is important for appropriate patient management.

**Case presentation:**

We describe here, two interesting cases of skin involvement in DLBCL- one primary cutaneous DLBCL and the other, cutaneous involvement in systemic DLBCL with different clinico-pathological profiles. Though both cases had almost similar morphology of the skin lesions (ulcero-proliferative) at presentation, the disease was confined to the skin in the former, while the latter had involvement of lymph nodes and bone marrow.

**Conclusions:**

Meticulous clinical evaluation, appropriate histopathological and immunohistochemical workup helped in their diagnosis and correct classification of the disease status, guiding the further treatment decisions.

## Background

Diffuse large B cell lymphoma (DLBCL), an aggressive tumour of mature B cells can occur in both lymphoid and extra-lymphoid locations and about 30–40% of cases primarily present in extra-nodal locations [1, 2]. Though gastrointestinal tract is the commonest of the extra-nodal sites, others include skin, bone, mediastinum, central nervous system, breast and others [1]. Skin involvement could be primary or secondary in patients with DLBCL. Depending on the primary site of involvement, DLBCL with cutaneous involvement can be divided into two groups—primary cutaneous DLBCL (PCDLBCL), which is confined to the skin, and DLBCL accompanied by secondary spread to the skin. Differentiating among these two types is essential for individual patient prognostication as they have different treatment and survival outcomes. Here in, we report two interesting cases of DLBCL presenting with skin involvement to start with, one diagnosed as PCDLBCL and other as systemic DLBCL with cutaneous involvement.

## Cases presentation

### Case 1

**Fig. 1:**
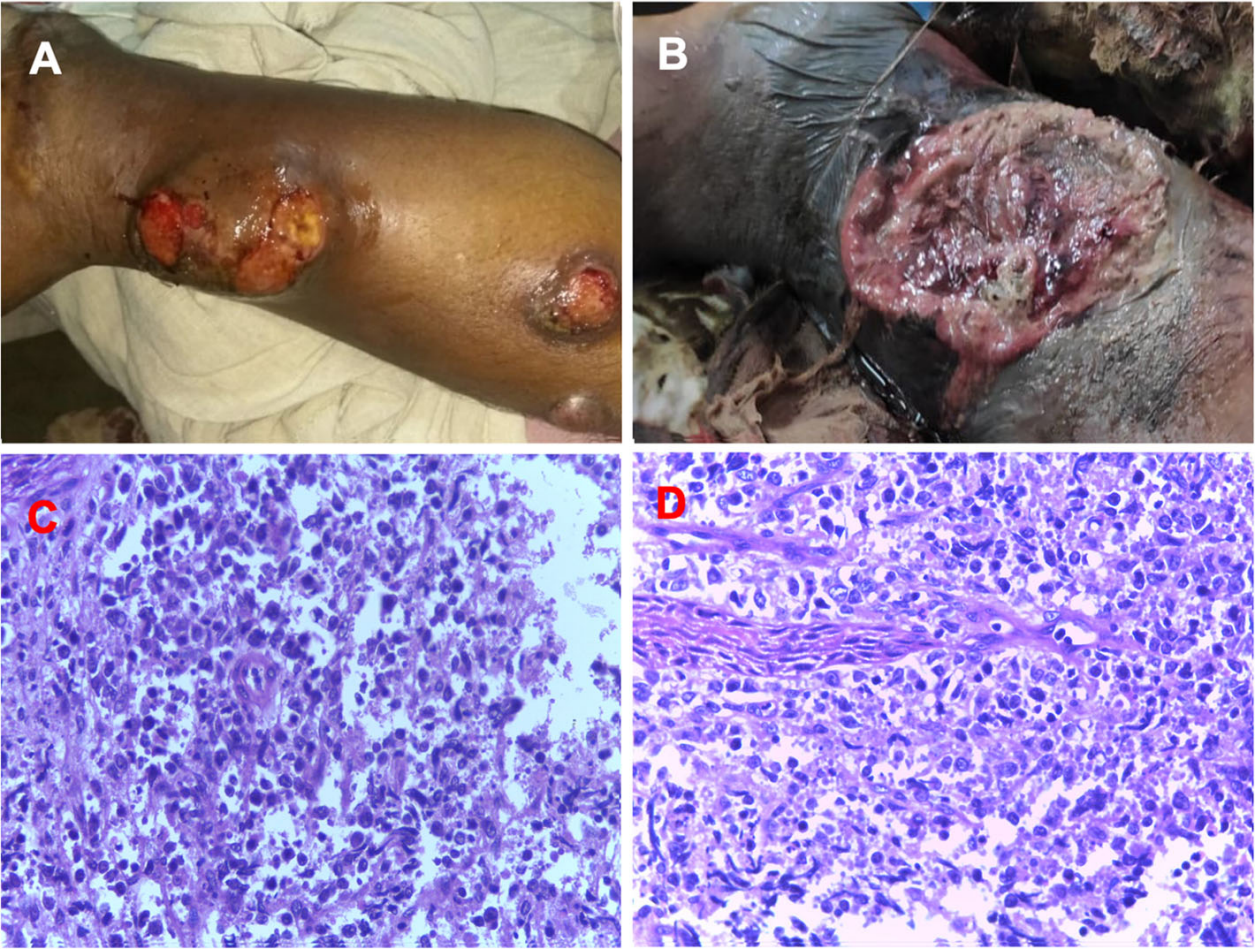
Cutaneous manifestations and histology of primary cutaneous diffuse large B cell lymphoma; **a** ulceroproliferative growth over the leg progressing to **b** gangrenous lesion; **c**, **d** H&E stained slides from the lesion showing diffuse infiltration by large atypical lymphoid cells with abundant cytoplasm

**Fig. 2:**
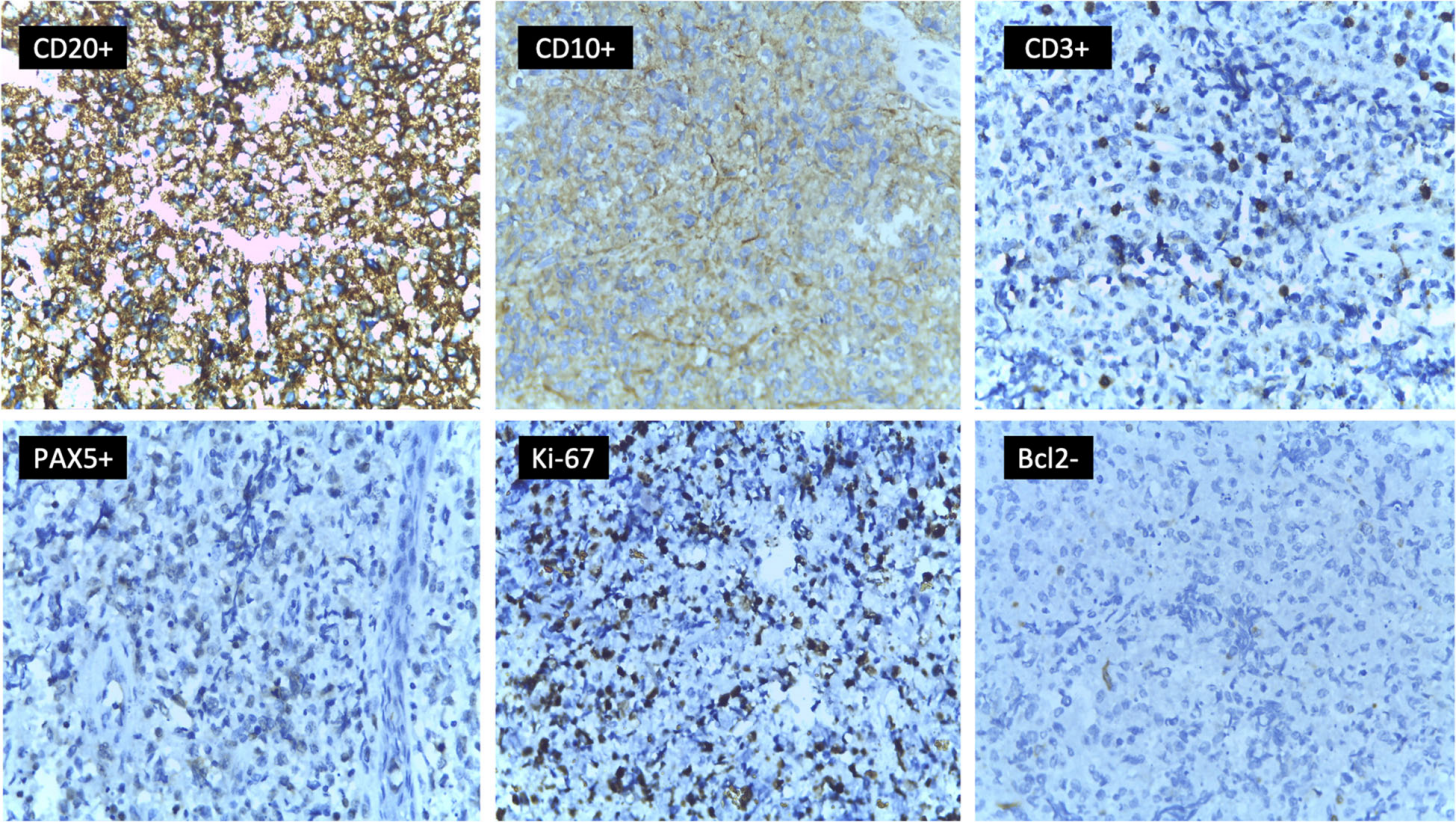
Immunohistochemistry showing the atypical cells positive for CD20, CD 10, CD3, PAX 5, Ki-67 index of > 60% and negative for bcl-2

A 72-year-old man, presented with a progressive ulcero-proliferative lesion over the left leg of 5 months duration (Fig. 1a, b). On examination, an ulcero-proliferative growth over an area of moist gangrenous swelling was noted from the medial epicondyle of the left leg till the tip of the toes. The skin was hyper-pigmented with multiple areas of ulceration and necrosis. The peripheral pulses were not palpable. Biopsy from the lesion showed diffuse infiltration of the dermis by large atypical lymphoid cells with abundant cytoplasm (Fig. 1c, d) and immunohistochemistry (IHC) was positive for CD20, CD 10, CD3, CD 5 and PAX 5 with a Ki-67 index of > 60% and negative for MUM-1, bcl-6 and bcl-2 (Fig. 2). Subsequently, total-body computed tomography (CT) did not show other disease localizations, so the diagnosis of primary cutaneous DLBCL germinal centre B cell (GCB) type was made. He received 3 cycles of R-CHOP (rituximab, cyclophosphamide, vincristine, prednisone) chemotherapy but in view of the progression of the lesion, an above knee amputation was performed. He had a recurrence at the stump site 8 months later and succumbed to his illness.

### Case 2

**Fig. 3:**
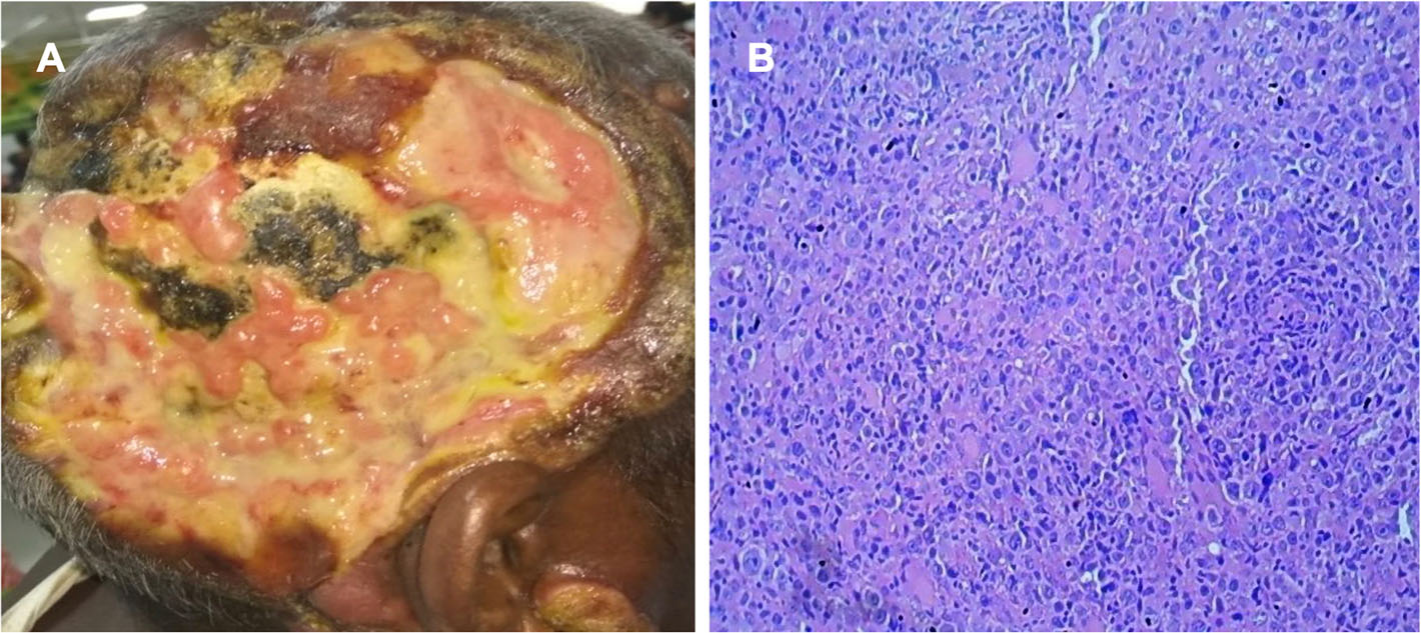
**a** Large ulcer over the right scalp; **b** H&E stained sections from the ulcerated lesion shows a diffuse population of atypical lymphoid cells

**Fig. 4:**
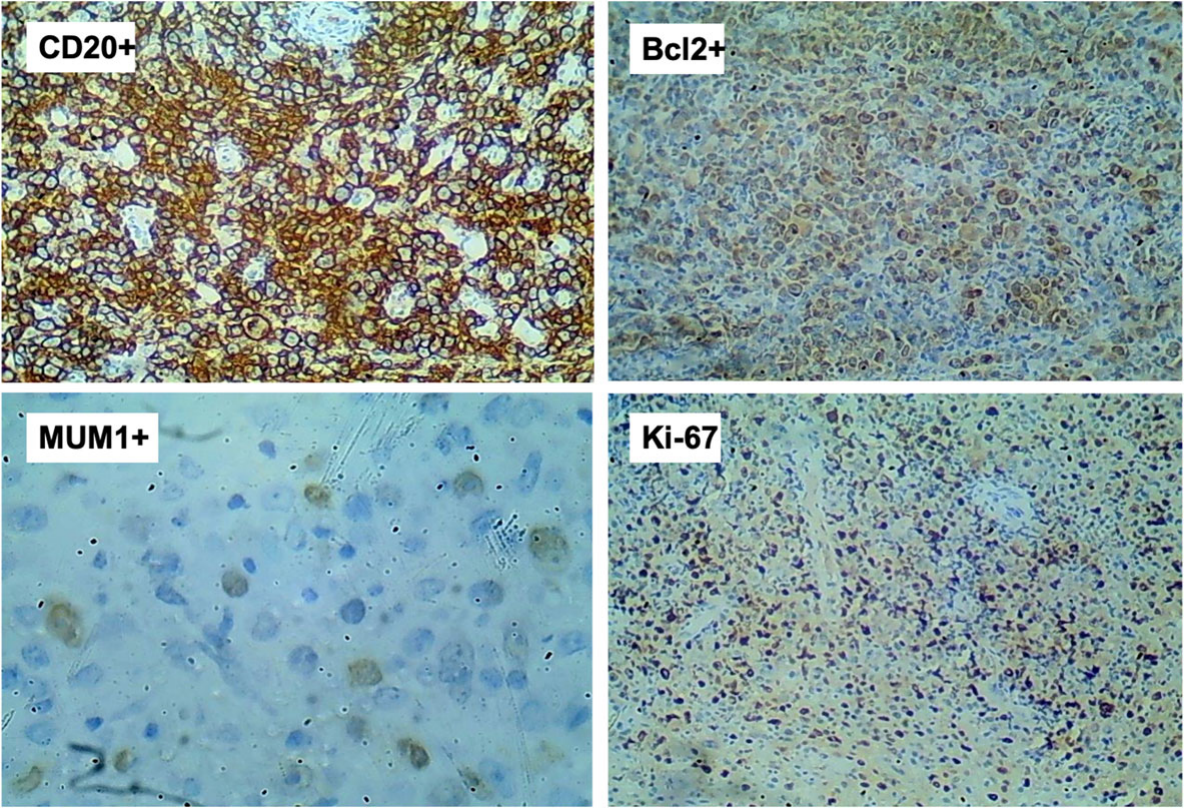
Immunohistochemistry of the atypical cells positive for CD 20, bcl-2, MUM-1 and Ki-67 index of 80%

**Fig. 5:**
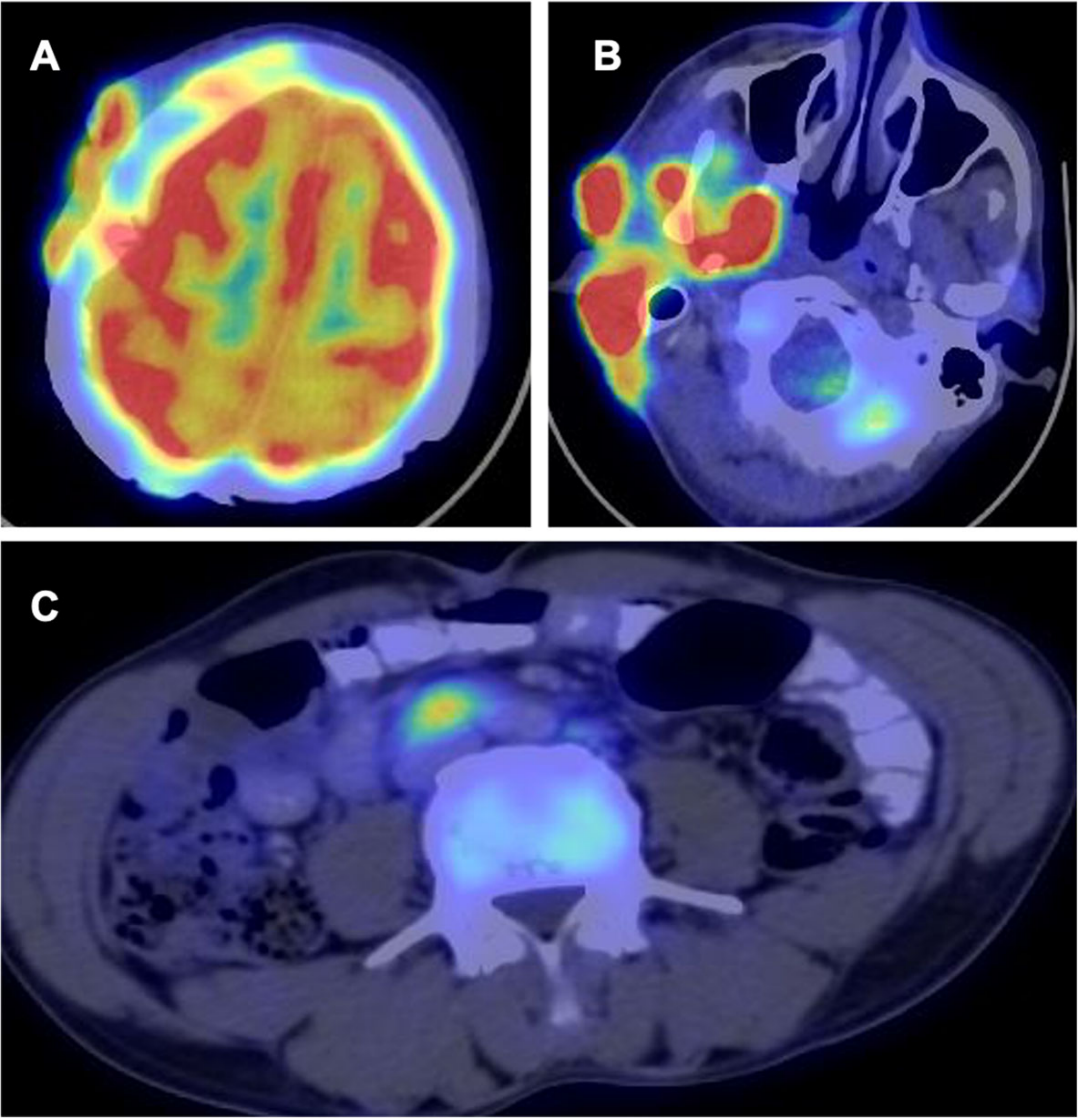
Positron emission tomography images of the ulcerated lesion. **a**, **b** Flanking the right frontal, parietal and temporo-occipital region, extending into the right infratemporal fossa, masseter and lateral pterygoid; **c** involvement of diaphragmatic lymph nodes

A 43-year-old man, presented with headache, vomiting for 1-week duration and a large ulcer over the right scalp measuring 20 × 20 cm covered with slough in base (Fig. 3a). Punch biopsy from the ulcerated lesion showed a diffuse population of lymphoid cells, round to oval, medium to large in size nucleus exhibiting fine chromatin, eosinophilic nucleoli with moderate pale cytoplasm (Fig. 3b). IHC was positive for CD 45, CD 20, bcl-2, bcl-6 and MUM-1 positive with a Ki-67 index of 80% and negative for CD 10, CD 3 and HMB 45 (Fig. 4). On further evaluation, bone marrow biopsy showed lymphomatous involvement. Cerebrospinal fluid (CSF) examination was negative for any malignant cells. Positron emission tomography (PET) was suggestive of ulcerated cutaneous thickening with ill-defined underlying subcutaneous soft tissue flanking the right frontal, parietal, temporo-occipital region, extending into the right infratemporal fossa, masseter and lateral pterygoid (Fig. 5). There was also the erosion of right skull bones with intracranial extension, narrowing of the jugular vein, involvement of diaphragmatic lymph nodes (both supra and infra) and bone marrow. Thus a final diagnosis of systemic DLBCL with stage 4 disease was made and he was started R-GDP (rituximab, gemcitabine, dexamethasone, cisplatin) chemotherapy (standard first-line CHOP was not given in view of his previous history of congestive cardiac failure). After completing 2 cycles of chemotherapy, he defaulted and was lost to follow-up.

## Discussion

The exact definition of primary extra-nodal DLBCL is debatable, particularly when both nodal and extra-nodal sites are involved [1, 3]. Phenotypic differences and single gene defects of c-MYC, bcl-6, REL and FOXP1 have been described for nodal and extra-nodal DLBCL [4, 5]. Clinical and pathological differences between nodal and extra-nodal DLBCL have been observed by many authors which, also translate into survival outcomes [1, 6, 7]. Among the extra-nodal sites, after the gastrointestinal tract, skin involvement predominates. In a study by Takahashi et al, skin involvement in DLBCL had significantly decreased the overall survival rate [8]. Primary cutaneous lymphomas tend to be indolent, whereas cutaneous involvement in systemic DLBCL, classifies the patient into a higher stage (Ann Arbor IV) leading to an unfavourable prognosis. Thus, identifying the clinical phenotypes with skin involvement is important both in the classification and management of patients as cutaneous involvement could be associated with a more aggressive course. It can be challenging to differentiate PCDLBCL from primary extra-nodal DLBCL presenting in the skin. While in our first case, the disease was limited to the skin and no extracutaneous spread was identified after evaluation, in the second case, primary extra-nodal presentation on the scalp was accompanied by involvement of the underlying skull bones, bone marrow and lymph nodes.

PCDLBCL constitutes 25–30% of primary cutaneous lymphomas [9] and can be divided into marginal zone B cell lymphoma, follicle centre lymphoma (PCFCL), PCDLBCL-leg type (LT). While PCDLBCL-LT show strong expression of bcl-2 as well as expression of MUM-1 and variable expression of bcl-6, those that do not show bcl-2 expression are characterised as PCDLBCL not otherwise specified (PCDLBCL-NOS). However, the role of bcl-2 expression in classifying these lymphomas is debatable and few researchers did not observe any difference in survival rates based on bcl-2 positivity [10]. Although the recent WHO classification [11] did not recognise the NOS variety as a separate entity, it has been described by many researchers and behaves differently compared to the follicular and leg type variants [12–14]. In a study by Felcht et al., the authors observed that the PCDLBCL-NOS tumours are composed more of CD3 infiltrate as was seen in our case [14]. A study of 161 cases of PCDLBCL with large-cell morphology by Lucioni et al. [15] showed that 25% of cases did not fulfil the criteria for either PCFCL or PCDLBCL-LT and were therefore classified as PCDLBCL-NOS. The authors also found that in the NOS variety, tumour cells were chiefly centroblasts intermingled with a variable reactive CD3+ lymphocytes and 27.5% were positive for CD10 while none of the LT lesions was CD10 positive. The classification system of PCDLBCL developed by Hans et al into germinal and non-germinal centre B cell like (GCB) subtypes bears an important effect of survival outcomes with non-GCB cases having poorer survival [16]. According to Hans algorithm, our first patient was classified as PCDLBCL-GCB subtype (bcl-6−; CD10+). The representation of T-lymphocytes in the lesion is useful in differentiating the types of PCDLBCL. While they are absent or very few in PCDLBCL-LT, they are better represented in PCDLBCL-NOS [17]. Distinction between GCB and non GCB subtypes is necessary as they tend to behave differently and have variable response rates to chemotherapy.

Primary extra-nodal DLBCL can be seen in up to 40% of cases. Skin involvement is less common and in a large cohort of DLBCL cases, skin involvement at presentation was seen in 3.3% of cases [7]. The morphology of skin lesions can be similar among the PCDLBCL and secondary cutaneous DLBCL. Non-contiguous involvement and extensive lesions however can be seen more often in secondary cutaneous DLBCL [18]. IHC alone cannot differentiate between a primary and systemic DLBCL. Majority of the DLBCL involving skin show activated B cell phenotype and express MUM1 and negative for CD10 as was seen in our case, although other combinations do exist [19]. If a proper evaluation for extracutaneous disease is not performed, DLBCL with skin involvement at presentation can be falsely labelled as PCDLBCL. As in our second case who presented with extensive skin lesion over the scalp, a meticulous evaluation identified extracutaneous disease and marrow involvement and thus a diagnosis of systemic DLBCL with cutaneous involvement could be made. As reported in literature, primary cutaneous lymphomas have a better prognosis [9, 10, 20] and outcomes with cutaneous involvement in systemic DLBCL could be variable. Some studies showed that patients with primary extra-nodal DLBCL had higher overall survival rates compared to primary nodal DLBCL [21, 22]. In the study by Lee et al., patients with systemic DLBCL and skin involvement had an advanced stage, higher IPI scores and poorer outcomes than PCDLBCL [18].

## Conclusions

To conclude, skin involvement at presentation in DLBCL could be either primary or secondary. Differentiating PCDLBCL from primary extra-nodal DLBCL presenting in the skin could be challenging at times. In this report, the first case was PCDLBCL-other, CD10+ GCB type and the second case was primary extra-nodal (skin) DLBCL with CD10−, and bcl-6, bcl-2, MUM 1 positivity. A meticulous evaluation for the search of extracutaneous disease is needed for correct classification of the disease, to initiate appropriate treatment regimens and importantly for patient prognostication and outcome measures.

## Data Availability

Not applicable.

## Abbreviations

DLBCL: Diffuse large B cell lymphoma
PCDLBCL: Primary cutaneous diffuse large B cell lymphoma
PCFCL: Primary cutaneous follicle centre lymphoma
IHC: Immunohistochemistry
CT: Computed tomography
PET: Positron emission tomography
CSF: Cerebrospinal fluid
GCB: Germinal centre B cell
